# The Comparative Analysis of Peptides in Enteral Nutrition Products and Foods for Special Medical Purposes

**DOI:** 10.3390/foods13162557

**Published:** 2024-08-16

**Authors:** Hao Li, Chenlu Fang, Yushan Hu, Jing Xu, Wei Zhao, Li Li

**Affiliations:** 1 State Key Laboratory of Food Science and Resources, Jiangnan University, Wuxi 214122, China; 2 School of Food Science and Technology, Jiangnan University, Wuxi 214122, China

**Keywords:** enteral nutrition, FSMPs, peptides, molecular weight distribution, peptide composition, bioactive peptide

## Abstract

Enteral nutrition (EN) and foods for special medical purposes (FSMPs) can be used to meet the specific nutritional needs of patients. There are multiple types of EN products and nutritionally complete FSMPs on the market. The peptides in these products are important nutritional components, while their presence in different products remains unclear. To provide better clinical guidelines, we analyzed and compared the molecular weight (MW) distribution, types, and quantities of peptides and bioactive peptides of two EN products (liquid products) and two FSMPs with nutritionally complete formulas (powder products). Our results showed that each product had a unique peptide profile. The two liquid products and one powder product (Samples 1–3) had a higher content of peptides. Sample 1 contained 75.60% peptides with an MW less than 375 Da and contained 95.21% peptides with an MW less than 1000 Da, being rich in short peptides. Sample 2 and 3 had high levels of peptides with MW values between 180 Da and 2000 Da. Additionally, Sample 4 contained high levels of proteins, containing 69.18% peptides with MW values larger than 10,000 Da. Further, Sample 1 had more bioactive dipeptides and Sample 2 had more long bioactive peptides. Our results suggest that peptides in different EN and FSMP products are very different and should be evaluated in more detail. This will provide valuable information for clinical medical professionals, help them to guide patients with different physiological conditions better, and ultimately benefit patients.

## 1. Introduction

Enteral nutrition (EN) formulas are specialized liquid nutritional products used to deliver essential nutrients directly into the gastrointestinal tract of patients who are unable to obtain adequate nutrition through oral intake. EN formulas include standard formulas and peptide-based formulas. Foods for special medical purposes (FSMPs) are foods that are specially processed and formulated to meet the special nutritional or dietary needs of individuals with restricted eating, digestive and absorption disorders, metabolic disorders, or specific disease statuses [1]. FSMPs include oral nutritional supplements and enteral tube-feeding products. Additionally, enteral nutritional suspensions with pharmaceutical registration are typically classified as FSMPs rather than drugs. EN products and FSMPs usually need to be consumed alone or in combination with other foods under the guidance of doctors or clinical nutritionists [2]. They can provide a comprehensive and balanced nutritional ratio, meet the needs of people with different health conditions, and offer advantages such as easy digestion and absorption. Additionally, EN products and FSMPs can offer clinical benefits and reduce healthcare costs by shortening hospital stays, promoting patient recovery and improving their life quality [3,4].

FSMPs are typically classified into several categories based on their intended use and nutritional composition, including nutritionally complete foods (for general use or disease specific), nutritionally incomplete foods (supplemental nutrition), modular products (individual nutrient components like protein, carbohydrate, fat, fiber, vitamins, and minerals) and enteral nutrition formulas (standard formulas and specialized formulas). Some studies have shown that certain EN formulas and FSMPs are beneficial for patients with specific diseases. Peptide-based EN formulas and FSMPs are suitable for patients with impaired digestive and absorption functions, food allergies, critical illnesses, severe malnutrition, and compromised pancreatic function or those suffering from conditions like Crohn’s disease and short bowel syndrome [5]. They display high bioavailability, low osmotic pressure, and good tolerance [6]. Short peptides can be more easily absorbed in the gastrointestinal tract, tend to emptied from the stomach more quickly, reduce allergic reactions, and improve tolerance compared to whole proteins. Moreover, they have low residue, require a small amount of digestive fluid to absorb, and produce a low amount of feces. By offering these benefits, short-peptide EN formulas and FSMPs play a critical role in supporting the nutritional needs and overall health of patients with specific medical conditions. However, different EN and FSMP products contain distinct peptide composition, and certain peptides have different metabolic characteristics and functions. Thus, we compared the peptide composition in two EN products and two FSMP products in this study, aiming to understand the EN and FSMP products available on the market better and provide important information for clinical medical professionals, enabling them to guide patients more accurately.

## 2. Materials and Methods

### 2.1. Materials

Two short-peptide-based EN products and two nutritionally complete FSMP products were purchased from online stores. The two enteral nutrition suspension products were defined as Sample 1 and Sample 2. The two FSMPs were powder products and could be taken orally or through a feeding tube, defined as Sample 3 and Sample 4. Sampling was carried out at various sales centers and across different batches. All products contained peptides derived from whey protein hydrolysates. Detailed information about the four products is shown in Table 1.

### 2.2. Measurement of Molecular Weight Distribution of Peptides by High-Performance Liquid Chromatography

The molecular weight (MW) distribution of the samples was determined using the HPLC (Waters 2695, Waters, Milford, MA, USA) equipped with the Waters 2487 ultraviolet detector. A 1% (*w*/*v*) solution of each sample was prepared and filtered through a microporous membrane, and then 10 μL of solution was injected into the HPLC system. The column used was the TSK gel 2000 SWXL (300 mm × 7.8 mm). The HPLC conditions were set as follows: the column temperature was 30 °C, the detection wavelength was 220 nm, the mobile phase was acetonitrile/water/TFA solution (40:60:0.1, *v*/*v*/*v*), and the flow rate was 0.5 mL/min. The standards used were Cytochrosme C (12384 Da; Sigma-Aldrich, Saint Louis, MO, USA), aprotinin (6500 Da; Sigma-Aldrich), bacitracin (1422 Da; Sigma-Aldrich), acetanine–acetanine–tyrosine–arginine (451 Da; Sigma-Aldrich), and acetanine–acetanine–acetanine (189 Da; Sigma-Aldrich).

### 2.3. Determination of Short Peptides by UHPLC-OE-MS Analysis

For liquid samples, 100 μL of each sample was taken and mixed with 400 μL of the extraction solution (MeOH:ACN, 1:1, *v*/*v*). For the powdered samples, 100 mg of each sample were taken and mixed with 500 μL of the extraction solution (MeOH:ACN:H_2_O, 2:2:1, *v*/*v*/*v*). The extraction solution contained deuterated internal standards. The mixed solution was vortexed for 30 s, sonicated at 4 °C for 10 min, and incubated at −40 °C for 1 h to precipitate proteins. Subsequently, the samples were centrifuged at 13,800× *g* at 4 °C for 15 min. The compounds in the supernatant were analyzed by the Vanquish ultra-high-performance liquid chromatography (UHPLC) system, coupled with an Orbitrap Exploris 120 mass spectrometer (Thermo Fisher Scientific, Waltham, MA, USA).

LC separation was performed by the Waters ACQUITY UPLC BEH Amideliquid chromatography column (2.1 mm × 50 mm, 1.7 μm). The mobile phase A consisted of 25 mmol/L ammonium acetate and 25 mmol/L ammonia hydroxide in water (pH = 9.75), and the mobile phase B was acetonitrile. The auto-sampler temperature was 4 °C and the injection volume was 2 μL. The MS/MS spectra were collected using the Orbitrap Exploris 120 mass spectrometer (Thermo Fisher Scientific, Waltham, MA, USA) set to information-dependent acquisition (IDA) mode under the control of the acquisition software (Xcalibur, version 4.4, Thermo). The detailed parameters are as follows: sheath gas flow rate of 50 Arb, Aux gas flow rate of 15 Arb, capillary temperature of 320 °C, full MS resolution of 60,000, MS/MS resolution of 15,000, collision energy of SNCE 20/30/40, and spray voltage of 3.8 kV (positive) or −3.4 kV (negative).

The raw data were converted into the mzXML format using the ProteoWizard software (V 3.0.24117), and metabolite identification was performed via a collaboratively written R package using BiotreeDB (V 3.0) database [7]. 

### 2.4. Peptide Determination by Nanolc-MS/MS Analysis

For sample preparation, 100 μL of each sample was taken and mixed with 400 μL of methanol, which was ultrasonicated in an ice-water bath for 5 min and then kept at −40 °C for 1 h. Next, the mixture was centrifuged at 13,800× *g* and 4 °C for 10 min, and 450 μL of the supernatant was collected and freeze-dried in a vacuum. Subsequently, the sample was dissolved in 200 μL of wash buffer and purified by a desalting column. Finally, 100 μL purified sample was collected and concentrated using a vacuum centrifugal concentrator, which was then resuspended in mobile phase A for later analysis.

For each sample, 200 ng of the total peptides was separated and analyzed using a nano UPLC (Evosep one) coupled to a timsTOF Pro2 instrument (Bruker) with a nano-electrospray ion source. LC separation was performed using a reverse-phase column (PePSep C18, 1.9 μm, 150 μm × 15 cm, Bruker, Germany). The mobile phases were H_2_O with 0.1% formic acid (FA) (phase A) and acetonitrile (ACN) with 0.1% FA (phase B). Sample separation was conducted with a 44 min gradient. The mass spectrometer was operated in data-dependent acquisition (DDA) PASEF mode, and the scanning range was from 100 to 1700 *m*/*z* for MS1. During PASEF MS/MS scanning, the impact energy increased linearly with ion mobility, from 20 eV (1/K_0_ = 0.6 Vs/cm^2^) to 59 eV (1/K_0_ = 1.6 Vs/cm^2^).

Vendor’s raw MS files were processed using the SpectroMine software (V 4.2.230428.52329) and the built-in Pulsar search engine. MS spectra lists were searched against their species-level UniProt FASTA databases (uniprot_Bos taurus_9913_reviewed_2023_11.fasta). Carbamidomethyl (C) was set as a fixed modification, and oxidation (M) and acetyl (protein N-term) were set as variable modifications. A maximum of 2 missed cleavages was allowed. The false discovery rate (FDR) was set to 0.01 for both peptide-spectrum-match (PSM) and peptide levels. Peptide identification was performed with an initial precursor mass deviation of up to 20 ppm and a fragment mass deviation of 20 ppm. The default settings were retained for all other parameters.

### 2.5. Statistical Analysis

Data were presented as mean ± standard deviation (SD) with three replicates (MW distribution analysis) or two replicates (peptide determination). All test data were analyzed using GraphPad Prism 8.0 (GraphPad Software Inc., San Diego, CA, USA). One-way analysis of variance (ANOVA) with Tukey’s test was used for comparison between groups. A *p*-value of less than 0.05 (*p* < 0.05) was considered significant in all analyses.

## 3. Results and Discussion

### 3.1. Molecular Weight Distribution of Peptides in Different EN and FSMP Products

**Figure 1:**
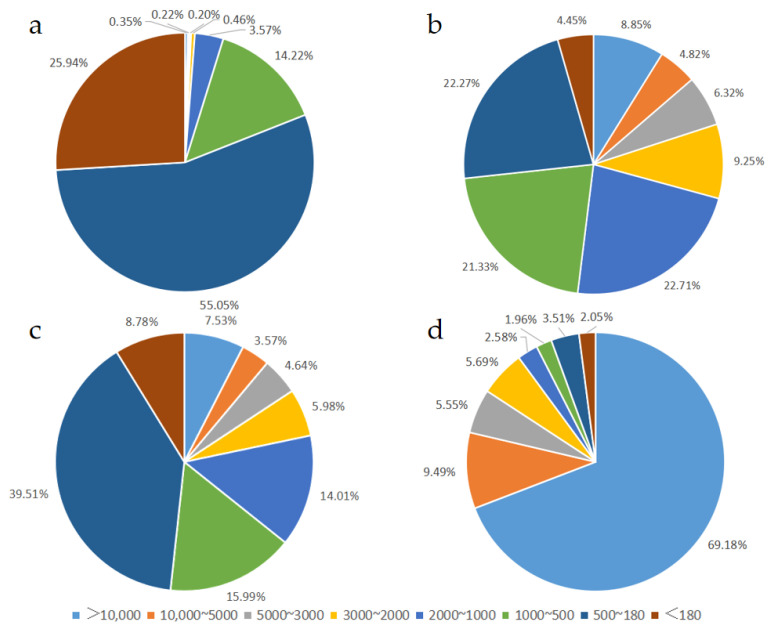
Molecular weight distribution of peptides: (**a**) Sample 1, (**b**) Sample 2, (**c**) Sample 3, (**d**) Sample 4. Range of >10,000, 10,000~5000, 5000~3000, 3000~2000, 2000~1000, 1000~500, 500~180, <180 Da was applied.

**Figure 2:**
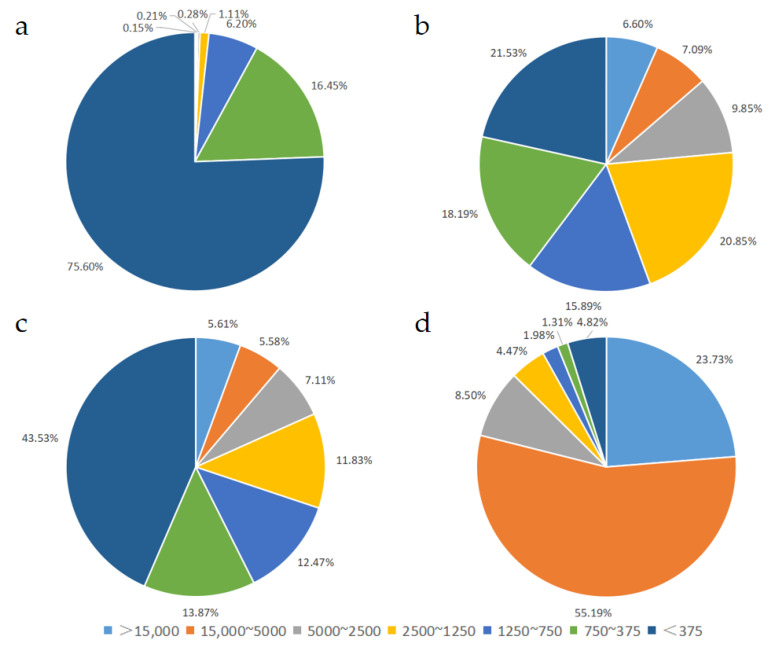
Molecular weight distribution of peptides: (**a**) Sample 1, (**b**) Sample 2, (**c**) Sample 3, (**d**) Sample 4. Range of >15,000, 15,000~5000, 5000~2500, 2500~1250, 1250~750, 750~375, <375 Da was applied.

To comprehensively evaluate the size distribution of peptides in each EN and FSMP product, the molecular weight distribution of peptides was determined by HPLC. The HPLC chromatogram of each sample is shown in Figures S1–S4. Two sets of ranges (>10,000, 10,000~5000, 5000~3000, 3000~2000, 2000~1000, 1000~500, 500~180, <180 Da; >15,000, 15,000~5000, 5000~2500, 2500~1250, 1250~750, 750~375, <375 Da) were applied in our study. Peptides with a MW of 180~1000 Da are commonly referred to as oligopeptides, and peptides with a MW larger than 1000 Da are referred to as polypeptides. Our results showed that the molecular size of peptides in each product was dramatically different (Figure 1 and Figure 2, Table 2, Table 3, Table 4 and Table 5). As shown in Figure 1 and Table 2, peptides with MW values of 500~180 Da occupied the highest proportion in Sample 1 and 3, taking up 55.05% and 39.51%, respectively. The peptides with MW values of 2000–1000, 1000–500, and 500–180 Da in Sample 2 were similar, accounting for 22.71%, 21.33%, and 22.27% of the samples, respectively. Additionally, the peptides with MW values higher than 10,000 Da had the highest proportion (69.18%) in Sample 4. Further, the proportion of peptides with MW values greater than 1000 in Sample 1 was very low, and the proportion of peptides with MW values less than 1000 was much higher than that seen in Samples 2, 3 and 4, which was up to 95.21%.

As shown in Figure 2 and Table 4, peptides with MW values lower than 375 Da and 375~750 Da in Sample 1 accounted for 75.60% and 16.45%, respectively. In Sample 2 and 3, peptides with MW values lower than 375 Da displayed the highest proportions, occupying 21.53% and 43.53% of the samples, respectively. Sample 4 had the highest proportion of peptides with MW values of 15,000~5000 Da (55.19%). The peptides with MW values less than 375 Da are usually dipeptides and tripeptides. Our results showed that Samples 1–3 had relatively high levels of dipeptides and tripeptides, and the levels of dipeptides and tripeptides in Sample 1 were much higher than that in Sample 2 and 3. As we can see, Sample 1 had the highest level of short peptides and oligopeptides, while Sample 4 had the lowest level of peptides. Moreover, the peptide MW values in each sample were distributed intensively within a narrow range, indicating that the molecular size of the peptides was evenly and continuously distributed.

It has been shown that the utilization efficiency and functional activity of peptides are closely related to their molecular weights. Low-molecular-weight peptides exhibit greater activity compared to high-molecular-weight peptides. This is primarily due to the fact that small peptides pass through intestinal epithelial cells easier, and then can be absorbed and transported to their targets effectively. Previous studies have shown that dipeptides and tripeptides can easily be absorbed intact by enterocytes with transporters such as PepT1 [5,8]. Moreover, oligomeric peptides with small molecular weight can be more readily absorbed by the human body without requiring additional energy during the absorption process, being friendly to the gastrointestinal tract [9]. Appropriate low-molecular-weight peptides showed high antioxidant activity [10,11,12]. Samaranayaka et al. observed that the molecular weight of most food-derived antioxidant peptides ranged from 500 to 1800 Da. Similarly, another study found that oligopeptides with molecular weights between 500 and 2000 Da exhibited higher antioxidant capacity compared to polypeptides [13]. The casein-derived peptides below 1000 Da exhibited the best initial and surviving antioxidant activities [14]. Additionally, short-peptide-based enteral nutrition can improve intestinal mucosal microcirculation, reduce mucosal inflammation, maintain the mechanical barrier and mucosal immunity, and defend commensal bacterial translocation in mice with severe acute pancreatitis [15].

### 3.2. Short Peptides with Less Than Five Amino Acids in Different EN and FSMP Products

**Figure 3:**
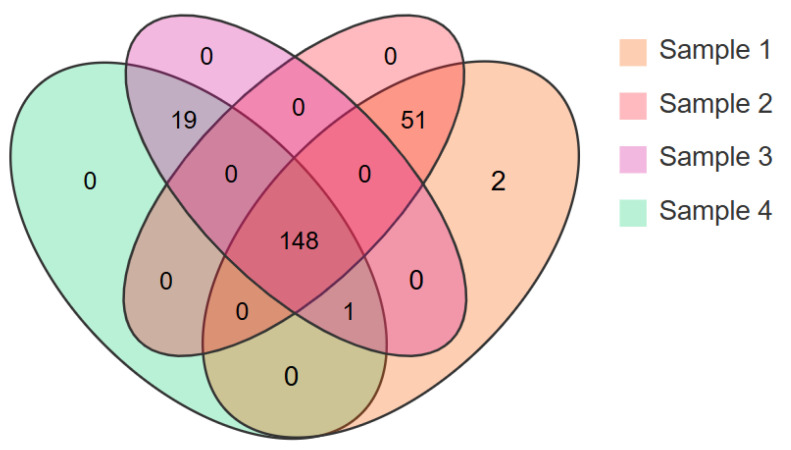
Venn diagram of amino acids and peptides in four EN and FSMP products. Number indicates types of amino acids and peptides detected in each sample.

To determine which types of peptides were present in each EN and FSMP product, the peptides in each product were analyzed by the UHPLC-OE-MS method. Many amino acids and short peptides with less than five amino acids were detected in the investigated products using this method. A total of 221 types of amino acids and peptides were detected in all samples (Table S1). According to Figure 3, 202 types of amino acids and peptides were detected in Sample 1, of which 183 types were peptides; 199 amino acids and peptides were detected in Sample 2, of which 180 types were peptides; 168 amino acids and peptides were detected in Sample 3, of which 152 types were peptides; and 168 amino acids and peptides were detected in Sample 4, of which 152 types were peptides. There were 148 types of amino acids and peptides detected in all four samples, and most of the detected peptides were dipeptides or tripeptides. The common amino acids and peptides present in all four samples included creatine, diglycine, Val-Ile, Leu-Ile, isoleucine, leucine and arginine. For example, creatine can be produced endogenously in the liver and kidney, and its supplementation has the effects of increasing exercise capacity and weight gain and promoting muscle protein synthesis [16,17]. The shared amino acids and peptides may provide basic nutritional support to individuals, and they may also impart specific functional properties to the products. In addition, Sample 1 and Sample 2 had more types of dipeptides and tripeptides. Previous research demonstrated that oligopeptides (>tetrapeptides) have a lower transport ability than dipeptides and tripeptides. Peptide length and the degradation of peptides by proteases may play a crucial role in the absorption process [18].

The top ten abundant amino acids and the top twenty abundant short peptides in each sample are shown in Table 6 and Table 7. The most abundant amino acids were as follows: isoleucine in Sample 1, leucine in Sample 2 and Sample 3, and proline in Sample 4 (Table 6). Further, as shown in Table S1 and Table 7, Sample 1 contained high levels of dipeptides, such as Leu-Ile, Val-Ile, Leu-Hpro, Val-Phe, and Leu-Val. The abundant peptides in Sample 2 included Val-Ile, Leu-Ile, Val-Tyr, Gly-Leu, and Leu-Phe; the abundant peptides in Sample 3 included Val-Phe, Leu-Leu, Val-Ile, Leu-Val, and Leu-Phe; and the most abundant peptides in Sample 4 included Val-Phe, Phe-Val, Val-Ile, Leu-Val, and Leu-Leu. Many dipeptides, like Val-Ile, Val-Phe, and Ile-Met, were found in all samples. Moreover, many peptides had higher abundance levels in Sample 1.

All samples contained taurine and gamma-Glutamylglutamic acid (γ-Glu-Glu). Samples 1 and 3 are rich in taurine, and the abundance of γ-Glu-Glu in Sample 1 is nearly 100 times higher than in Sample 2. Taurine is an essential nutrient in certain species and a semi-essential nutrient in humans. It has various physiological functions and can improve clinical and nutritional health in humans [19,20]. For example, taurine can enhance immunity and plays a key role in treating muscle-, central nervous system-, cardiovascular system- and metabolic system-related diseases [20]. Based on extensive animal and human studies, taurine has been approved as a therapeutic agent in the treatment of heart failure in Japan [21]. Its beneficial effects for patients with congestive heart failure may be due to a reduction in oxidative stress and calcium overload, mitigating neurohumoral-mediated cellular hypertrophy and the progression of overt heart failure [21]. Research has shown that γ-Glu-Glu exhibits numerous biological activities. γ-Glu-Glu can enhance glutamate responses on NMDA receptors and have excitatory effects on neurons. It was discovered that the concentration of extracellular γ-Glu-Glu is somewhat directly linked to glutathione (GSH) metabolism, as γ-Glu-Glu can be produced from the breakdown of GSH following the action of γ-glutamyl transferase [22]. These facts suggest that γ-Glu-Glu may have immunomodulatory and antioxidant properties. In the food industry, γ-Glu-Glu is widely used as a flavor enhancer, for fragrance, and as a seasoning agent.

### 3.3. Peptides with More Than Seven Amino Acids in Different EN Products

**Figure 4:**
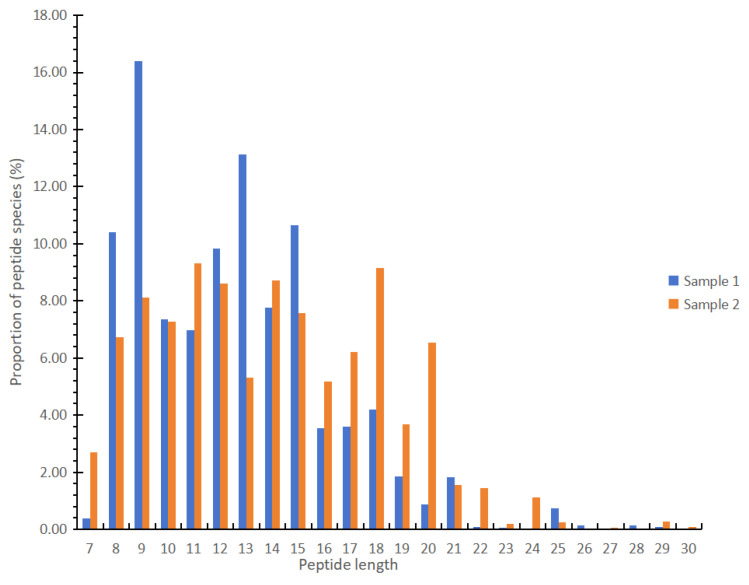
The length distribution of peptides in EN products.

Through the analysis of peptide molecular weight distribution, we found that two liquid EN products (Sample 1 and Sample 2) and one powdered FSMP product (Sample 3) had many oligopeptides and polypeptides. Moreover, the short-peptide analysis showed that Sample 1 and Sample 2 had more short peptides. Thus, we specifically examined the peptides in Sample 1 and Sample 2 using another method (nanoLC-MS/MS analysis). The method can detect peptides with more than five amino acids. The peptides found in each sample are shown in Table S2. The results showed that 308 peptides, exhibiting a degree of polymerization of 7–30 amino acids, were identified in Sample 1, and 305 peptides were identified in Sample 2. As shown in Figure 4, the peptides in both samples were mainly concentrated within the range of seven to twenty-two amino acids. In Sample 1, the proportion of peptides containing nine amino acids was the highest, reaching 16.39%, while in Sample 2, the proportion of peptides containing eleven amino acids was the highest, reaching 9.31%. The proportion of octapeptides, nonapeptides, tridecapeptides and pentadecapeptides in Sample 1 was significantly higher than that in Sample 2. In sample 2, most peptides were composed of 8–20 amino acids.

The top twenty abundant peptides in each sample are shown in Table 8. The most abundant peptides in Sample 1 included YPFPGPIP (8), YPFPGPIPN (9), KEMPFPKYP (9), KPTPEGDLE (9) and APKHKEMPFPKYP (13). While the most abundant peptides in Sample 2 included VEELKPTPEGDLEILLQK (18), VYVEELKPTPEGDLEILLQK (20), VEELKPTPEGDLEIL (15), VYVEELKPTPEGDLEIL (17), and LVLDTDYKK (9). As can be seen from Table 9, most of the peptides in Sample 1 and 2 are derived from beta-lactoglobulin and beta-casein, and some of these peptides showed high abundance. Beta-lactoglobulin is the main whey protein in the milk of ruminants and some non-ruminants (such as cows and horses) but it does not exist in human milk. Its function may be related to the transport of hydrophobic small molecules such as fatty acids and retinol [23]. Beta-lactoglobulin is the main allergen in infant milk allergies. This is because the infant’s digestive system is not fully developed, allowing beta-lactoglobulin to be adsorbed onto the intestinal mucosa, triggering an immune response and causing allergies.

The highest level of peptide YPFPGPIP present in Sample 1 was from beta-casein. Beta-casein is widely found in the milk of mammals (cows, goats, etc.) and human beings. It is a phosphorylated protein synthesized by mammary acinar epithelial cells. Beta-casein molecules contain one or more phosphorylated threonine or serine residues, which are structurally close to each other, and can chelate calcium, keeping calcium ions in a soluble state. This promotes calcium absorption, which is beneficial for infant growth and development [24].

Some peptides with high abundance in Sample 1 were from kappa-casein, which is one of phosphoproteins containing a small amount of phosphate in milk. In addition to the nutritional function of milk protein, kappa-casein hydrolysis can also release an antithrombotic dodecapeptide, which can inhibit ADP-induced platelet agglutination or fibrinogen binding process. That is, it has the effect of inhibiting blood coagulation and preventing thrombosis [25]. Studies have shown that the production of glucose-containing peptides after the enzymatic hydrolysis of kappa-casein can stimulate the growth of bifidobacteria in the intestinal tract of newborns and inhibit *Escherichia coli* formation, which is conducive to improving the digestive tract health of infants and children and preventing diarrhea [26].

### 3.4. Bioactive Peptides in Different EN and FSMP Products

Most peptides containing 2 to 20 amino acid residues have biological functions, such as antioxidative, anti-inflammatory, antimicrobial, antihypertensive, and anticancer effects [27,28]. Thus, the bioactive peptides in EN and FSMP products were examined in our study. For short peptides with less than five amino acids, we found 112, 110, 87, and 87 bioactive peptides in Sample 1, 2, 3, and 4, respectively (Table S3). The detected bioactive peptides with high abundance levels included Val-Phe, Leu-Leu, Phe-Val, Gly-Leu, etc. (Table 10). Further, we found 8 and 11 bioactive peptides with more than seven amino acids in Sample 1 and 2 (Table 11), including YPFPGPIP, YPFPGPIPN, VEELKPTPEGDLEIL, and VLVLDTDYK. Our results showed that Sample 1 had more types and levels of short bioactive peptides, while Sample 2 had more types of long bioactive peptides. These bioactive peptides offer the advantages of efficient absorption and diverse functions. They can directly bind to specific receptors in the body, accurately regulating physiological processes.

The most abundant bioactive peptides were Leu-Ile, Leu-Hpro, Val-Phe and Leu-Val in Sample 1; Leu-Ile, Val-Tyr, Gly-Leu and Leu-Phe in Sample 2; Val-Phe, Leu-Leu, Leu-Val and Leu-Phe in Sample 3; and Val-Phe, Phe-Val, Leu-Val and Leu-Leu in Sample 4. Previous studies have shown that three dipeptides isolated from corn peptides—Val-Leu, Leu-Leu and Leu-Gln—can promote protein synthesis, inhibit protein degradation, and increase protein accumulation in skeletal muscle by regulating the Akt-mTOR and NF-κB signaling pathways, suggesting their important roles in promoting the skeletal muscle growth [29]. Four BCAA-containing dipeptides (Val-Leu, Leu-Val, Ile-Leu, and Leu-Ile) obtained from whey protein hydrolysates modulate important biological parameters following acute exercise, showing anti-stress effects, attenuating exercise-induced immunosuppression, stimulating glycogen content and plasma glutamine levels, and beingbeneficial for protein synthesis and tissue protection [30]. However, our understanding about the function of certain bioactive peptides remains limited, and this should be studied in more detail in the future.

Our results showed that there were significant differences in the types and abundances of peptides and bioactive peptides in different products. Based on the distribution characteristics of peptides in different products, accurate clinical guidance can be provided. For example, Sample 1 has a higher proportion of short peptides, which are easily absorbed. It is recommended for individuals with digestion and absorption disorders. Sample 4 has a higher protein content, being suitable for patients with protein malnutrition or hypoproteinemia but with normal intestinal digestion and absorption functions. Additionally, bioactive peptides have functions such as being anti-inflammatory, being antioxidant, offering immune enhancement, and promoting muscle growth. According to the types, levels, and specific functions of bioactive peptides in different products, precise nutritional guidance can also be provided for different people: patients with enteritis, immunocompromised patients, or long-term bedridden patients.

## 4. Conclusions

To better understand the nutrients in different EN and FSMP products and provide better clinical guidelines, we compared the molecular weight distribution of peptides and assessed the peptide and bioactive peptide composition in two EN products and two FSMP products with nutritionally complete formulas. The results showed significant differences in the molecular weight distribution and peptide and bioactive peptide composition among the products. These differences may impact the absorption efficiency and biological activity of nutrients. Sample 1 is rich in short peptides and bioactive dipeptides, and these peptides can be easily absorbed by the human body and are friendly to the gastrointestinal tract. Sample 2 and Sample 3 contain high levels of peptides with MW values between 180 Da and 2000 Da, and Sample 2 has more types of long bioactive peptides. Sample 4 contains high levels of protein. Compared with the two liquid products, the two powdered products have fewer types of bioactive peptides. Our study is a preliminary exploratory investigation, and our results suggest that peptides in different EN and FSMP products are different and should be evaluated in more detail. By comprehensively comparing the peptide characteristics of different enteral nutritional suspensions and FSMP products, clinical medical professionals can be provided with more comprehensive and authoritative theoretical support. This will enable them to guide patients more accurately, optimize treatment plans, and improve patient nutritional status and overall treatment effectiveness.

## Figures and Tables

**Table 1:** Information for the four EN and FSMP products.

	Energy Supply Ratio	Product Classification	Product Status	Approval Number
Sample 1	Protein: 16% Carbohydrate: 69% Fat: 15%	Enteral nutrition suspension (SP)	Liquid	H2***5
Sample 2	Protein: 18% Carbohydrate: 57% Fat: 25%	Enteral nutrition emulsion (SP)	Liquid	H2***4
Sample 3	Protein: 14% Carbohydrate: 68% Fat: 18%	Whole Nutritional Formula Food for Special Medical Purposes	Powder	TY2***7
Sample 4	Protein: 19% Carbohydrate: 47% Fat: 34%	Whole Nutritional Formula Food for Special Medical Purposes	Powder	TY2***5

***: in order to protect the sample information, several numbers in the approval number sequence have been replaced with asterisks.

**Table 2:** Molecular weight analysis of peptides in each sample. Range of >10,000, 10,000~5000, 5000~3000, 3000~2000, 2000~1000, 1000~500, 500~180, <180 Da was applied.

	Relative Molecular Mass Distribution	Retention Time of Each Peak/Min	Peak Area Percentage/%	Number -Average Molecular Weight	Weight -Average Molecular Weight
Sample 1	>10,000	12.088	0.35 ± 0.61	17,810	20,482
	10,000~5000	13.558	0.22 ± 0.35	6050	6216
	5000~3000	14.589	0.20 ± 0.21	3631	3695
	3000~2000	15.483	0.46 ± 0.08	2508	2729
	2000~1000	16.736	3.57 ± 0.14	1218	1263
	1000~500	17.864	14.22 ± 1.01	636	662
	500~180	19.247	55.05 ± 4.85	251	267
	<180	19.800	25.94 ± 6.49	94	113
Sample 2	>10,000	11.517	8.85 ± 2.77	16,119	17,729
	10,000~5000	13.890	4.82 ± 0.15	6801	7091
	5000~3000	14.797	6.32 ± 0.08	3734	3815
	3000~2000	15.515	9.25 ± 0.10	2387	2419
	2000~1000	15.762	22.71 ± 1.57	1385	1442
	1000~500	17.520	21.33 ± 0.46	694	719
	500~180	18.695	22.27 ± 3.03	313	329
	<180	20.365	4.45 ± 0.91	98	114
Sample 3	>10,000	11.740	7.53 ± 1.08	15,531	16,328
	10,000~5000	13.890	3.57 ± 0.05	6788	7068
	5000~3000	14.797	4.64 ± 0.24	3727	3807
	3000~2000	15.410	5.98 ± 0.18	2405	2437
	2000~1000	16.714	14.01 ± 0.28	1342	1406
	1000~500	16.745	15.99 ± 0.49	691	722
	500~180	19.108	39.51 ± 0.53	266	281
	<180	19.786	8.78 ± 1.43	86	112
Sample 4	>10,000	12.015	69.18 ± 2.08	14,543	15,454
	10,000~5000	12.682	9.49 ± 2.46	8249	8520
	5000~3000	14.620	5.55 ± 0.64	3498	3551
	3000~2000	14.891	5.69 ± 0.20	2527	2558
	2000~1000	15.553	2.58 ± 0.22	1426	1495
	1000~500	16.791	1.96 ± 0.08	697	729
	500~180	19.139	3.51 ± 0.11	278	294
	<180	20.454	2.05 ± 0.30	99	109

**Table 3:** The differences of molecular weight of peptides in each sample. Range of >10,000, 10,000~5000, 5000~3000, 3000~2000, 2000~1000, 1000~500, 500~180, <180 Da was applied.

Relative Molecular Mass Distribution	<180	500~180	1000~500	2000~1000	3000~2000	5000~3000	10,000~5000	>10,000
Sample 1	25.94 ± 6.49 ^a^	55.05 ± 4.85^a^	14.22 ± 1.01 ^c^	3.57 ± 0.14 ^c^	0.46 ± 0.08 ^c^	0.20 ± 0.21 ^d^	0.22 ± 0.35 ^c^	0.35 ± 0.61 ^c^
Sample 2	4.45 ± 0.91 ^bc^	22.27 ± 3.03 ^c^	21.33 ± 0.46 ^a^	22.71 ± 1.57 ^a^	9.25 ± 0.10 ^a^	6.32 ± 0.08 ^a^	4.82 ± 0.15 ^b^	8.85 ± 2.77 ^b^
Sample 3	8.78 ± 1.43 ^b^	39.51 ± 0.53 ^b^	15.99 ± 0.49 ^b^	14.01 ± 0.28 ^b^	5.98 ± 0.18 ^b^	4.64 ± 0.24 ^c^	3.57 ± 0.05 ^b^	7.53 ± 1.08 ^b^
Sample 4	2.05 ± 0.30 ^c^	3.51 ± 0.11 ^d^	1.96 ± 0.08 ^d^	2.58 ± 0.22 ^c^	5.69 ± 0.20 ^b^	5.55 ± 0.64 ^b^	9.49 ± 2.46 ^a^	69.18 ± 2.08 ^a^

Different letters in the same column indicate significant differences (*p* < 0.05); results were the average values of three parallel experiments.

**Table 4:** Molecular weight analysis of peptides in each sample. Range of >15,000, 15,000~5000, 5000~2500, 2500~1250, 1250~750, 750~375, <375 Da was applied.

	Relative Molecular Mass Distribution	Retention Time of Each Peak/Min	Peak Area Percentage/%	Number -Average Molecular Weight	Weight -Average Molecular Weight
Sample 1	>15,000	10.857	0.15 ± 0.25	22,467	23,865
	15,000~5000	12.876	0.21 ± 0.34	6904	7364
	5000~2500	14.633	0.28 ± 0.24	4225	4345
	2500~1250	15.943	1.11 ± 0.84	2087	2157
	1250~750	16.742	6.20 ± 0.68	946	962
	750~375	17.864	16.45 ± 0.76	525	540
	<375	19.247	75.60 ± 1.58	156	201
Sample 2	>15,000	11.517	6.60 ± 0.85	18,741	19,933
	15,000~5000	12.586	7.09 ± 2.01	7826	8570
	5000~2500	15.121	9.85 ± 0.16	3294	3426
	2500~1250	15.762	20.85 ± 0.86	1712	1776
	1250~750	16.560	15.89 ± 1.20	967	989
	750~375	17.520	18.19 ± 1.28	550	571
	<375	18.695	21.53 ± 3.34	209	260
Sample 3	>15,000	11.740	5.61 ± 0.61	17,107	17,681
	15,000~5000	12.584	5.58 ± 1.69	7980	8781
	5000~2500	15.121	7.11 ± 0.31	3310	3439
	2500~1250	15.711	11.83 ± 0.08	1742	1807
	1250~750	16.714	12.47 ± 0.15	968	987
	750~375	17.708	13.87 ± 0.24	537	557
	<375	19.108	43.53 ± 1.14	198	235
Sample 4	>15,000	11.797	23.73 ± 3.11	18,269	19,115
	15,000~5000	11.863	55.19 ± 3.15	11,562	12,117
	5000~2500	14.513	8.50 ± 0.10	3152	3233
	2500~1250	15.024	4.47 ± 0.52	1949	2019
	1250~750	16.665	1.98 ± 0.55	908	939
	750~375	17.775	1.31 ± 0.67	500	514
	<375	19.089	4.82 ± 0.53	170	209

**Table 5:** The differences in molecular weight of the peptides in each sample. Range of >15,000, 15,000~5000, 5000~2500, 2500~1250, 1250~750, 750~375, <375 Da was applied.

Relative Molecular Mass Distribution	<375	750~375	1250~750	2500~1250	5000~2500	15,000~5000	>15,000
Sample 1	75.60 ± 1.58 ^a^	16.45 ± 0.76 ^b^	6.20 ± 0.68 ^c^	1.11 ± 0.84 ^d^	0.28 ± 0.24 ^d^	0.21 ± 0.34 ^c^	0.15 ± 0.25 ^c^
Sample 2	21.53 ± 3.34 ^c^	18.20 ± 1.28 ^a^	15.89 ± 1.20 ^a^	20.85 ± 0.86 ^a^	9.85 ± 0.16 ^a^	7.09 ± 2.01 ^b^	6.60 ± 0.85 ^b^
Sample 3	43.53 ± 1.14 ^b^	13.87 ± 0.24 ^c^	12.47 ± 0.15 ^b^	11.83 ± 0.08 ^b^	7.11 ± 0.31 ^c^	5.58 ± 1.69 ^b^	5.61 ± 0.61 ^b^
Sample 4	4.82 ± 0.53 ^d^	1.31 ± 0.67 ^d^	1.98 ± 0.55 ^d^	4.47 ± 0.52 ^c^	8.50 ± 0.10 ^b^	55.19 ± 3.15 ^a^	23.72 ± 3.11 ^a^

Different letters in the same column indicate significant differences (*p* < 0.05); all results were the average values of three parallel experiments.

**Table 6:** The top 10 amino acids in each EN or FSMP product.

Sample 1	Sample 2	Sample 3	Sample 4
Isoleucine	Leucine	Leucine	Proline
Leucine	Arginine	Isoleucine	Leucine
Valine	Lysine	Valine	Isoleucine
Phenylalanine	Isoleucine	Phenylalanine	Arginine
Arginine	Proline	Methionine	Lysine
Lysine	Phenylalanine	Taurine	Phenylalanine
Proline	Valine	Proline	Histidine
Threonine	Tyrosine	Histidine	Valine
Homoserine	Asparagine	Threonine	Methionine
Methionine	Methionine	Homoserine	Tyrosine

**Table 7:** The top 20 most abundant short peptides in each EN or FSMP product.

Sample 1	Sample 2	Sample 3	Sample 4
Leu-Ile	Val-Ile	Val-Phe	Val-Phe
Val-Ile	Leu-Ile	Leu-Leu	Phe-Val
Leu-Hpro	Val-Tyr	Val-Ile	Val-Ile
Val-Phe	Gly-Leu	Leu-Val	Leu-Val
Leu-Val	Leu-Phe	Leu-Phe	Leu-Leu
Ile-Met	Phe-Leu	Phe-Leu	Leu-Phe
Leu-Leu	Tyr-Val	Phe-Val	Phe-Leu
Ile-Leu	Leu-Val	Ile-Ala	Val-Tyr
Ile-Asp	Pro-Phe	Leu-Ala	Ile-Ala
Phe-Val	Thr-Val-Leu	Ile-Met	Ile-Ser
Ile-Trp	Ile-Trp	Tyr-Ile	Ile-Met
Leu-Ala	Leu-Leu	Ile-Ser	Isoleucyl-Threonine
Leu-Glu	Ile-Leu	Gly-Leu	Creatine
Ile-Ser	Maculosin	Isoleucyl -Phenylalanine	Ala-Leu
Ala-Leu	Val-Phe	Val-Ala	Anorexigenic Peptide
Gly-Leu	Thr-Lys	Isoleucyl-Threonine	Leu-Ala
Tyr-Ile	Ile-Met	Val-Tyr	Tyr-Val
Gly-Gly-Leu	Val-Lys	Thr-Leu	Val-Ala
Val-Tyr	Leu-Gly	Gly-Gly-Leu	Ile-Gly-Ile
Val-Ala	Ala-Leu	Tyr-Leu	Gly-Leu

**Table 8:** The top 20 most abundant peptides with more than seven amino acids in the two liquid EN products. The content proportion represents the ratio of peptide abundance in all detected peptides.

	Protein Descriptions	Peptide Stripped Sequence	Peptide Length	Content Proportion/%
Sample 1	Beta-casein	YPFPGPIP	8	6.58
	Beta-casein	YPFPGPIPN	9	5.98
	Beta-casein	KEMPFPKYP	9	4.89
	Beta-lactoglobulin	KPTPEGDLE	9	3.95
	Beta-casein	APKHKEMPFPKYP	13	3.89
	Beta-casein	KEMPFPKY	8	2.04
	Kappa-casein	AIPPKKNQDKTEIPT	15	1.88
	Zinc-alpha-2 -glycoprotein	SKPREGFPSFQAV	13	1.85
	Zinc-alpha-2 -glycoprotein	PAAQNTKRKWEAEAVYVQ	18	1.68
	Glycosylation -dependent cell adhesion molecule 1	SRQPQSQNPKLPLSILK	17	1.57
	Beta-lactoglobulin	KGLDIQKVAGTWYSL	15	1.49
	Kappa-casein	IPPKKNQDKTEIPT	14	1.46
	Beta-2-microglobulin	VTLEQPRIVKW	11	1.39
	Beta-2-microglobulin	IQRPPKIQVY	10	1.22
	Beta-2-microglobulin	VTLEQPRIVKWD	12	1.13
	Beta-lactoglobulin	LKPTPEGDLE	10	1.09
	Beta-lactoglobulin	DISLLDAQSAPLRVY	15	1.00
	Xanthine dehydrogenase/oxidase	PTYTSATLLFQKD	13	0.99
	Polymeric immunoglobulin receptor	KSPIFGPEEVTSVEGRSVSIK	21	0.96
	Beta-lactoglobulin	VEELKPTPEGDLEIL	15	0.95
Sample 2	Beta-lactoglobulin	VEELKPTPEGDLEILLQK	18	4.93
	Beta-lactoglobulin	VYVEELKPTPEGDLEILLQK	20	4.76
	Beta-lactoglobulin	VEELKPTPEGDLEIL	15	2.88
	Beta-lactoglobulin	VYVEELKPTPEGDLEIL	17	2.48
	Beta-lactoglobulin	LVLDTDYKK	9	2.40
	Beta-lactoglobulin	SDISLLDAQSAPLR	14	2.39
	Beta-lactoglobulin	VLVLDTDYKK	10	1.94
	Beta-lactoglobulin	VLDTDYKK	8	1.56
	Beta-lactoglobulin	VLVLDTDYK	9	1.55
	Beta-lactoglobulin	LDTDYKK	7	1.54
	Beta-lactoglobulin	VEELKPTPEGDLEILLQ	17	1.32
	Beta-lactoglobulin	SLLDAQSAPLR	11	1.29
	Beta-lactoglobulin	EVDDEALEKFDK	12	1.29
	Beta-lactoglobulin	SLAMAASDISLLDAQSAPLR	20	1.25
	Beta-lactoglobulin	TPEVDDEALEK	11	1.23
	Beta-lactoglobulin	LIVTQTMK	8	1.22
	Glycosylation -dependent cell adhesion molecule 1	ILNKPEDETHLEAQPTDASAQF	22	1.21
	Beta-lactoglobulin	TPEVDDEALEKFDK	14	1.21
	Beta-lactoglobulin	VLDTDYK	7	1.15
	Beta-lactoglobulin	SLAMAASDISLL	12	1.01

**Table 9:** The number of peptides from certain source proteins and their content. The content proportion represents the ratio of peptide abundance in all detected peptides.

Sample	Protein Descriptions	Peptide Quantity	Content Proportion/%
Sample 1	Beta-casein	14	25.89
	Beta-lactoglobulin	71	20.49
	Zinc-alpha-2-glycoprotein	24	9.90
	Xanthine dehydrogenase/oxidase	51	9.49
	Glycosylation-dependent cell adhesion molecule 1	18	6.13
	Beta-2-microglobulin	5	4.13
	Kappa-casein	7	3.89
	Butyrophilin subfamily 1 member A1	11	1.95
Sample 2	Beta-lactoglobulin	99	63.08
	Beta-casein	32	7.18
	Glycosylation-dependent cell adhesion molecule 1	34	6.85
	Alpha-S1-casein	13	4.66
	Albumin	16	2.75
	Alpha-lactalbumin	7	2.16
	Prostaglandin-H2 D-isomerase	6	1.48
	Nucleobindin-1	10	1.26

**Table 10:** The top 20 most abundant bioactive peptides with less than five amino acids in four EN and FSMP products.

Sample 1	Sample 2	Sample 3	Sample 4
Leu-Ile	Leu-Ile	Val-Phe	Val-Phe
Leu-Hpro	Val-Tyr	Leu-Leu	Phe-Val
Val-Phe	Gly-Leu	Leu-Val	Leu-Val
Leu-Val	Leu-Phe	Leu-Phe	Leu-Leu
Ile-Leu	Phe-Leu	Phe-Leu	Leu-Phe
Leu-Leu	Tyr-Val	Phe-Val	Phe-Leu
Phe-Val	Leu-Val	Leu-Ala	Val-Tyr
Ile-Trp	Pro-Phe	Tyr-Ile	Creatine
Leu-Ala	Thr-Val-Leu	Gly-Leu	Ala-Leu
Ala-Leu	Ile-Trp	Val-Ala	Leu-Ala
Gly-Leu	Ile-Leu	Val-Tyr	Tyr-Val
Tyr-Ile	Leu-Leu	Tyr-Leu	Val-Ala
Val-Tyr	Maculosin	Val-Glu	Gly-Leu
Val-Ala	Val-Phe	Thr-Val-Leu	Gly-Pro-Arg-Pro -amide
Leu-Phe	Leu-Gly	Tyr-Trp	Tyr-Ile
Phe-Leu	Ala-Leu	Pro-Met	Tyr-Trp
Thr-Val-Leu	Tyr-Ile	Phe-Met	Leu-Trp
Val-Met	Leu-Trp	Tyr-Val	Thr-Val-Leu
Tyr-Val	(3xi,6xi)-Cyclo (alanylvalyl)	Ala-Leu	Phe-Met-Arg-Phe -amide
Ile-Pro-Ile	Val-Ala	Leu-Asp	Met-Thr

**Table 11:** The bioactive peptides with more than seven amino acids in the two EN products.

Bioactive Peptide Sequence				
Sample 1	YPFPGPIP	YPFPGPIPN	LKPTPEGDLE	VEELKPTPEGDLEIL
	LKPTPEGDLEIL	ELKPTPEGDLEIL	TQTPVVVPPF	LVRTPEVDDE
Sample 2	VEELKPTPEGDLEIL	VLVLDTDYK	TPEVDDEALEK	VLDTDYK
	LDAQSAPLR	PFPEVFGK	FFVAPFPEVFGK	ELKPTPEGDLEIL
	YLGYLEQLLR	HQPHQPLPPTVMFPPQ	RELEELNVPGEIVESLSSSEESITR	

## Data Availability

The original contributions presented in the study are included in the article/Supplementary Materials, further inquiries can be directed to the corresponding author.

## Supplementary Materials

The following supporting information can be downloaded at https://www.mdpi.com/article/10.3390/foods13162557/s1. The supporting data can be seen in Figures S1–S4 and Tables S1–S3. Figures S1–S4: The chromatograms of samples for the analysis of the molecular weight distribution of peptides. Table S1: The amino acids and peptides detected in EN and FSMP products. Table S2: Peptides with more than seven amino acids detected in EN products. Table S3: Bioactive peptides with less than five amino acids in different EN and FSMP products.
