# Supplementary material for: The Comparative Analysis of Peptides in Enteral Nutrition Products and Foods for Special Medical Purposes
Source: Foods. 2024 Aug 16;13(16):2557. doi: 10.3390/foods13162557 (PMC11353486; doi:10.3390/foods13162557)
Supplement: Supplementary file 1 [file foods-13-02557-s001.zip › Figure S1-4.pdf]

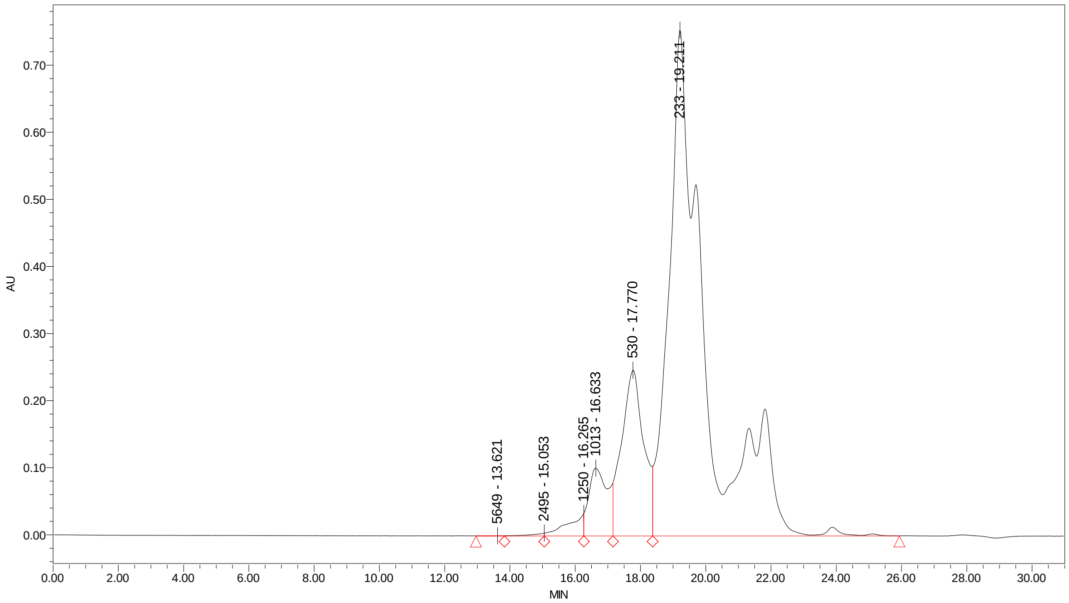

Figure S1. Chromatogram of Sample 1.

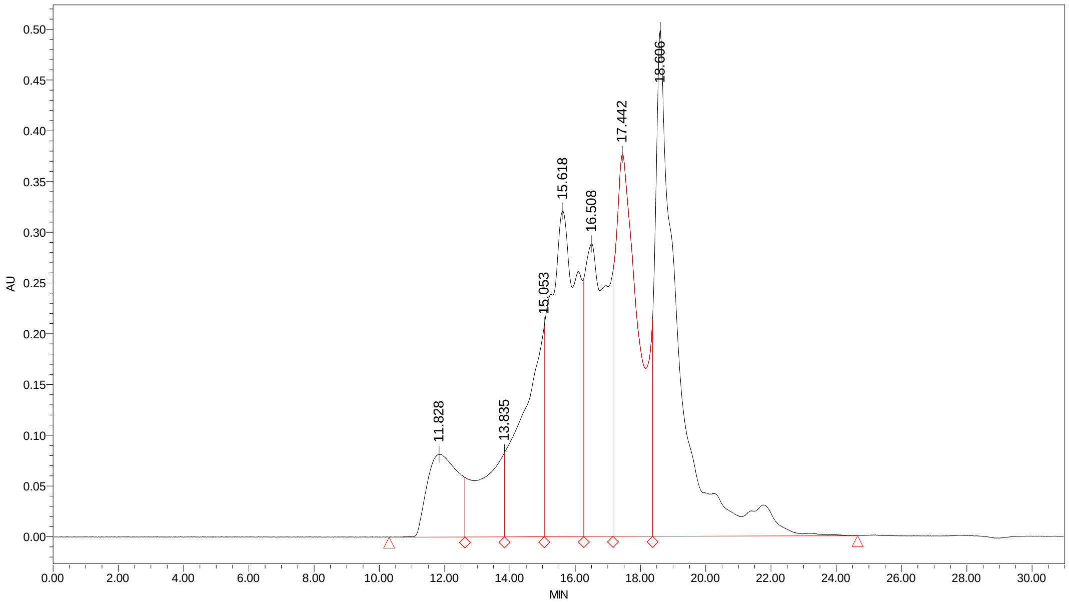

Figure S2. Chromatogram of Sample 2.

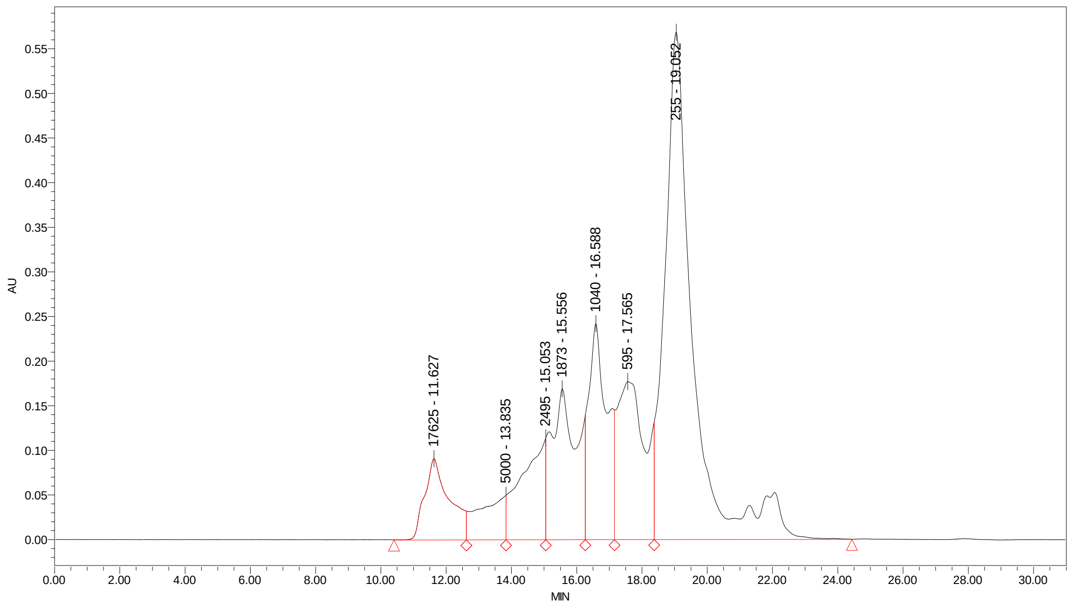

Figure S3. Chromatogram of Sample 3.

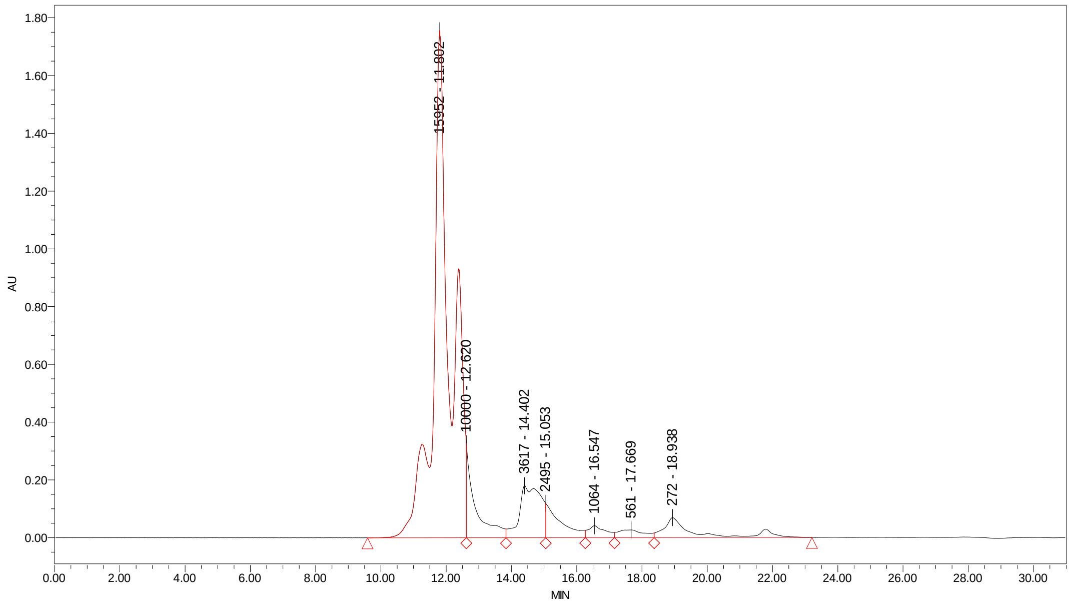

Figure S4. Chromatogram of Sample 4.
